# 823. Clinical Outcomes of Coccidioidomycosis in Patients Requiring Intensive Care Unit Hospitalization

**DOI:** 10.1093/ofid/ofad500.868

**Published:** 2023-11-27

**Authors:** James Ray M Lim, Ashley Scott, Rebecca Wig, Rachel V Tan, Emily R Harnois, Tirdad Zangeneh, Mohanad Al-Obaidi

**Affiliations:** Cambridge Health Alliance, Melrose, Massachusetts; University of Arizona, Tucson, Arizona; University of Arizona, College of Medicine, Tucson, tucson, Arizona; University of Arizona - Tucson, Tucson, Arizona; University of Arizona College of Medicine-Tucson, Tucson, Arizona; University of Arizona, Tucson, Arizona; University of Arizona, Tucson, Arizona

## Abstract

**Background:**

Coccidioidomycosis is an endemic mycosis in the southwestern United States. While most infections are mild or asymptomatic, severe cases are associated with high morbidity and mortality. We aimed to evaluate the clinical outcomes of patients admitted to the intensive care unit (ICU) with proven culture-positive coccidioidomycosis.

**Methods:**

After institutional review board approval, we retrospectively included ICU patients with positive *Coccidioides* spp. culture within a multi-healthcare system between 10/01/2017 – 07/01/2022. Clinical information was collected by chart review.

**Results:**

Of the 392 hospital encounters with positive *Coccidioides* spp. culture, 145 patients required ICU stay. The median age was 51 (32-62), with 100 (68.9%) male, 56 (38.6%) White non-Hispanics, and 25 (17.2%) Blacks. Fifty-seven (39.3%) received azole antifungals before hospitalization. Seventy-two (49.7%) had another fungal, bacterial, or viral infection during their hospitalization, and 41 (28.3%) had extrapulmonary coccidioidomycosis. The majority, 131 (90.3%), received antifungal therapy during their hospital stay. The median hospital length of stay was 18 days (10-37). Almost half (48.3%) died during the study period, with liver cirrhosis and age ( >60 years) resulting in statistically significant mortality, 12/13 (92.3%), p-value < 0.001, and 31/43 (72.1%) p-value =0.001, respectively. Mechanical ventilation and/or intravenous vasopressor support was associated with a high risk of mortality 66/113 (58.4%, p-value=< 0.001). Failure to receive antifungal therapy before ICU admission was associated with increased mortality, 46/70 (65.7%, p-value=0.002).

**Conclusion:**

Patients with coccidioidomycosis admitted to the ICU face an increased risk of mortality. Risk factors associated with ICU mortality include advanced age, cirrhosis, and delay in the administration of antifungal therapy. Future studies are needed to evaluate these risks further.

**Disclosures:**

**Tirdad Zangeneh, DO**, AiCuris: Grant/Research Support **Mohanad Al-Obaidi, MD, MPH**, La Jolla Pharmaceuticals: Honoraria

